# Supplementary material for: Identification of CD318, TSPAN8 and CD66c as target candidates for CAR T cell based immunotherapy of pancreatic adenocarcinoma
Source: Nat Commun. 2021 Mar 5;12:1453. doi: 10.1038/s41467-021-21774-4 (PMC7935963; doi:10.1038/s41467-021-21774-4)
Supplement: Supplementary file 1 — Supplementary information [file 41467_2021_21774_MOESM1_ESM.pdf]

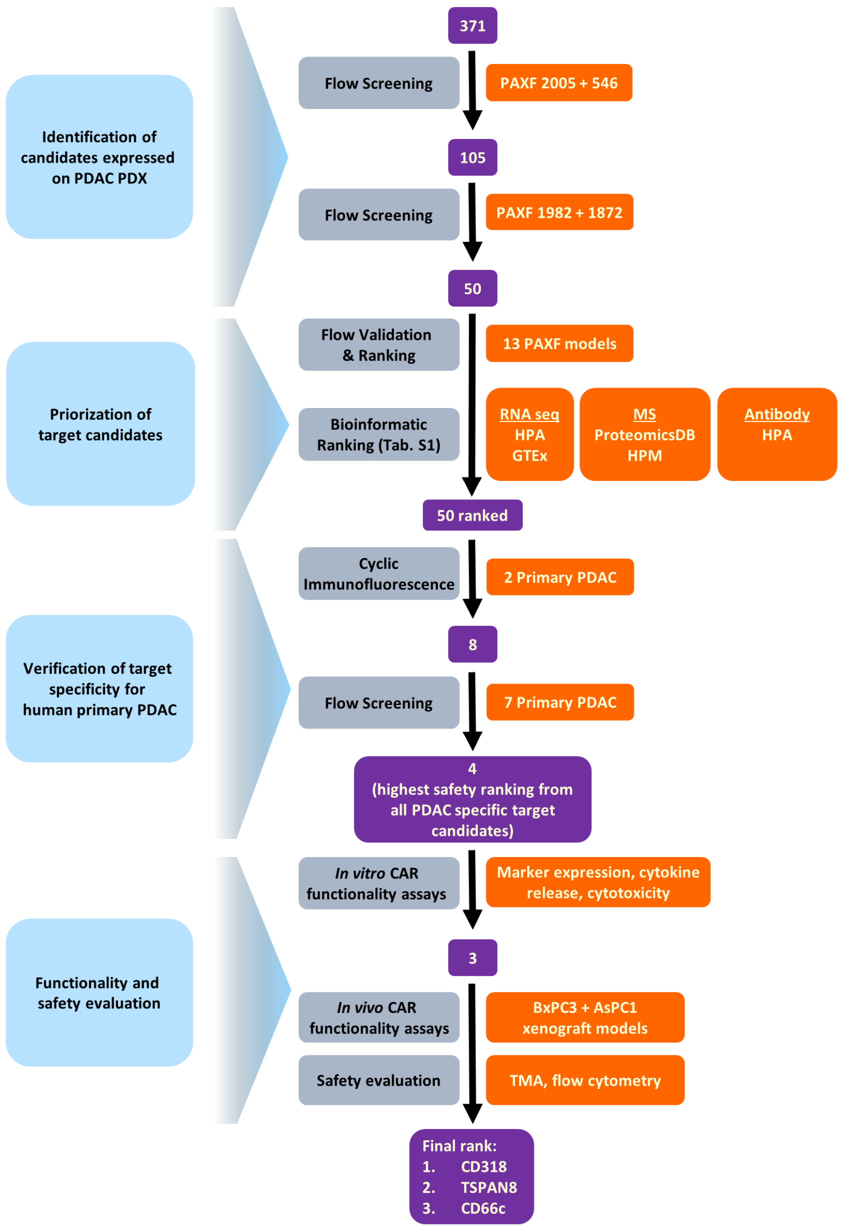

Supplementary Fig. 1: Target discovery workflow.

Scheme of the workflow for identification of target candidates as applied in this study.

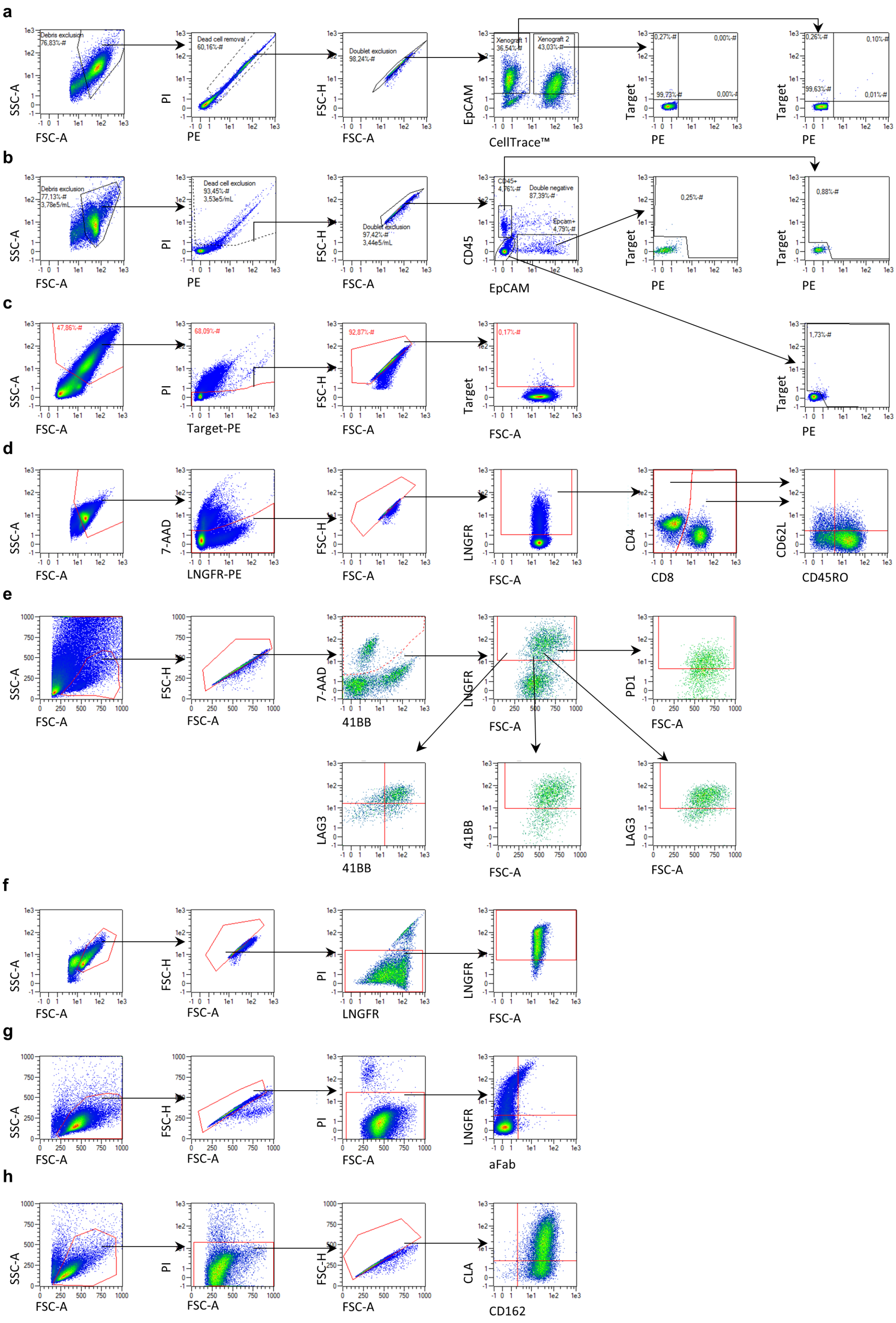
a
Debris exclusion
76,83%-#
Dead cell removal
60,16%-#
Doublet exclusion
98,24%-#
Xenograft 1
36,54%-#
Xenograft 2
43,03%-#
SSC-A
FSC-A
PI
PE
FSC-H
EpCAM
CellTrace™
Target
b
Debris exclusion
77,13%-#
3,78e5/mL
Dead cell exclusion
93,45%-#
3,53e5/mL
Doublet exclusion
97,42%-#
3,44e5/mL
CD45+
4,76%-#
Double negative
87,39%-#
Epcam+
4,79%-#
0,25%-#
0,88%-#
CD45
EpCAM
c
47,86%-#
68,09%-#
92,87%-#
0,17%-#
1,73%-#
Target-PE
d
7-AAD
LNGFR-PE
LNGFR
CD4
CD8
CD62L
CD45RO
e
41BB
PD1
LAG3
f
g
aFab
h
CLA
CD162

Supplementary Figure 2: Gating strategies for flow cytometric analyses.

(a) Gating strategy for evaluation of target expression on dissociated patient derived xenograft models of pancreatic ductal adenocarcinoma (PDAC) presented on Supplementary Fig. 1a. **(b)** Gating strategy for evaluation of target expression on primary human PDAC presented on Supplementary Fig. 1c, Supplementary Supplementary Fig. 4a. **(c)** Gating strategy for evaluation of target expression on dissociated xenografts or PDAC cell lines presented in Supplementary Fig. 2b, 5d, Supplementary Supplementary Fig. 5a,b, Supplementary Supplementary Fig. 11. **(d)** Gating strategy for analysis of *in vivo* CAR T cell phenotype and cell count presented in Supplementary Fig. 5a,b, Supplementary Fig. 7a,b. **(e)** Gating strategy for analysis of CAR T cell activation marker expression presented in Supplementary Fig. 3b,d, Supplementary Fig. 8a, Supplementary Supplementary Fig. 7b, Supplementary Supplementary Fig. 8a,c. **(f)** Gating strategy for CAR transfection analysis presented in Supplementary Supplementary Fig. 6. **(g)** Gating strategy for reporter protein and CAR expression on T cells depicted in Supplementary Supplementary Fig. 9c. **(h)** Gating strategy for analysis of CLA and CD162 expression on T cells presented in Supplementary Supplementary Fig. 9a,c.

a

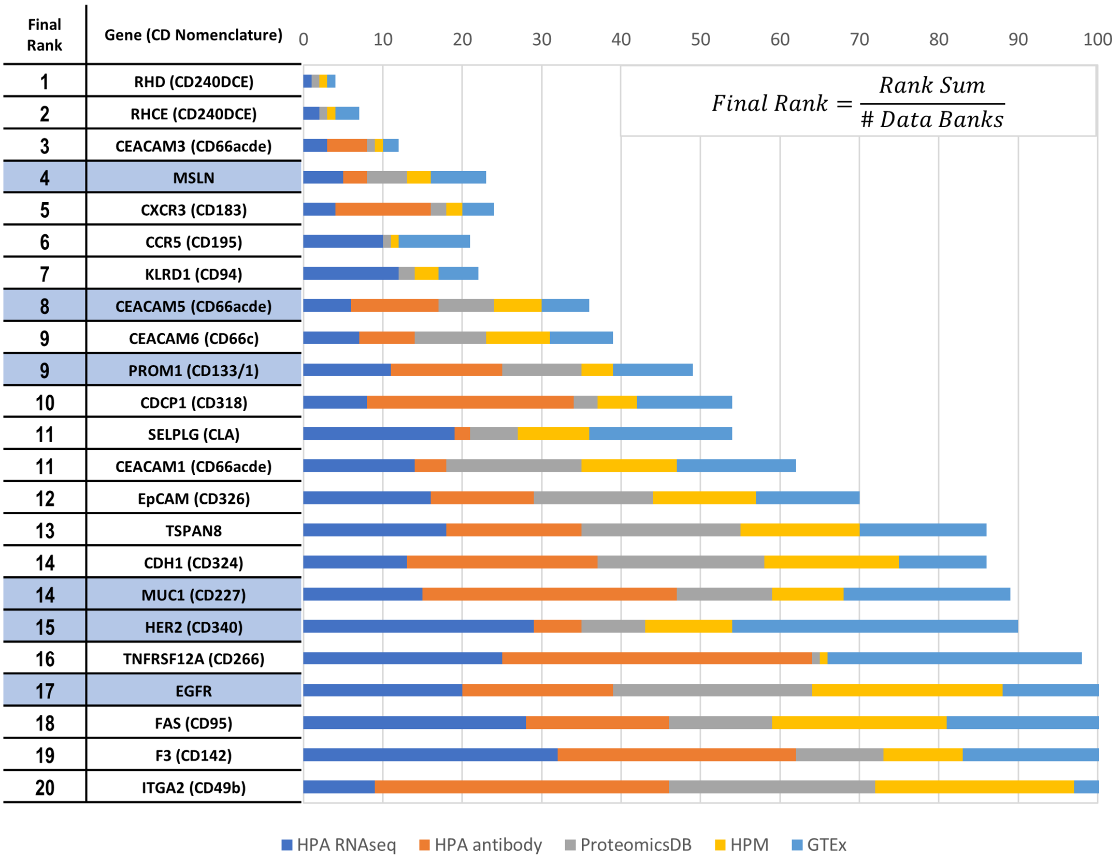

b

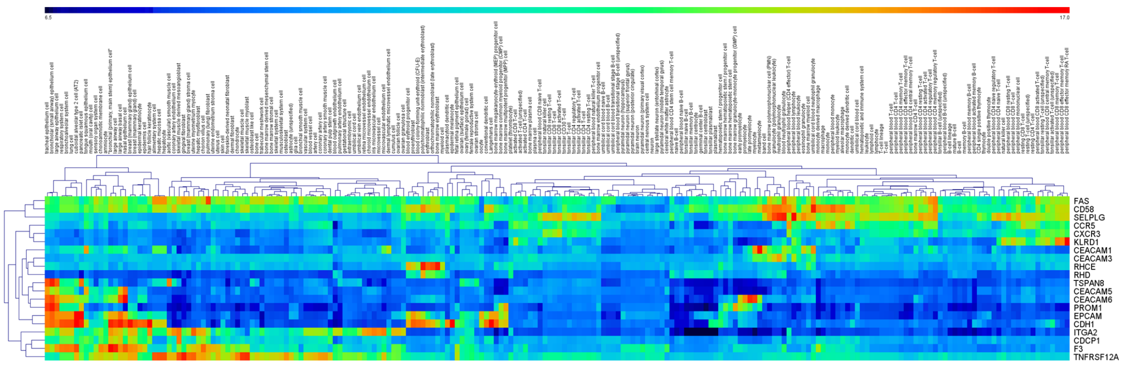

Supplementary Fig. 3: Bioinformatics based ranking of target candidates.

(a) 20 most promising candidate genes ranked by the quotient of the rank sum and number of data sources the respective gene was found in. The ranking also includes targets currently under clinical investigation which

appeared in our ranking but were not included in the initial antibody array or failed to meet inclusion criteria during the PDX screening (blue filling). Final rank with lowest off-tumor expression at the top; HPA RNAseq or antibody = Human Protein Atlas mRNA or antibody-based protein expression, HPM = Human Proteome Map, GTEx = Genotype-Tissue Expression. **(b)** Heatmap of the expression of 20 most promising genes in healthy tissues (data source: Genevestigator). Source data are provided as a Source Data file.

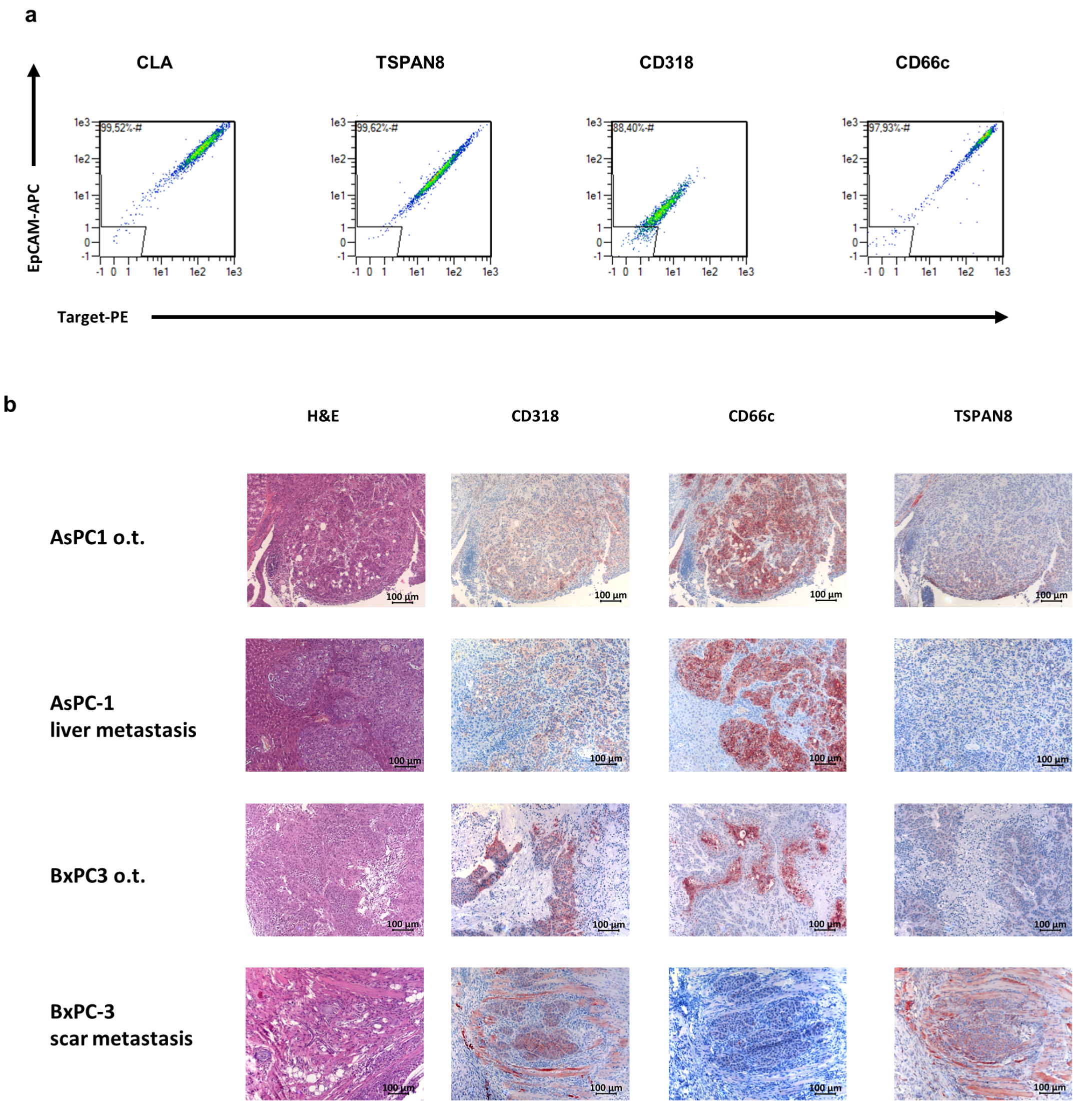

Supplementary Fig. 4: Analysis of target candidate expression on primary human PDAC and o.t. and metastatic xenograft models.

(a) Representative density plots of CLA, TSPAN8, CD318 and CD66c expression on primary PDAC. The summarized data from all 8 primary human tumors can be found in Supplementary Fig. 1c. **(b)** H&E and IHC stainings of CLA, TSPAN8, CD318 and CD66c expression on tumors from orthotopically transplanted PDAC cell lines AsPC1 and BxPC3 and metastases thereof. Images are representative for tumor triplicates.

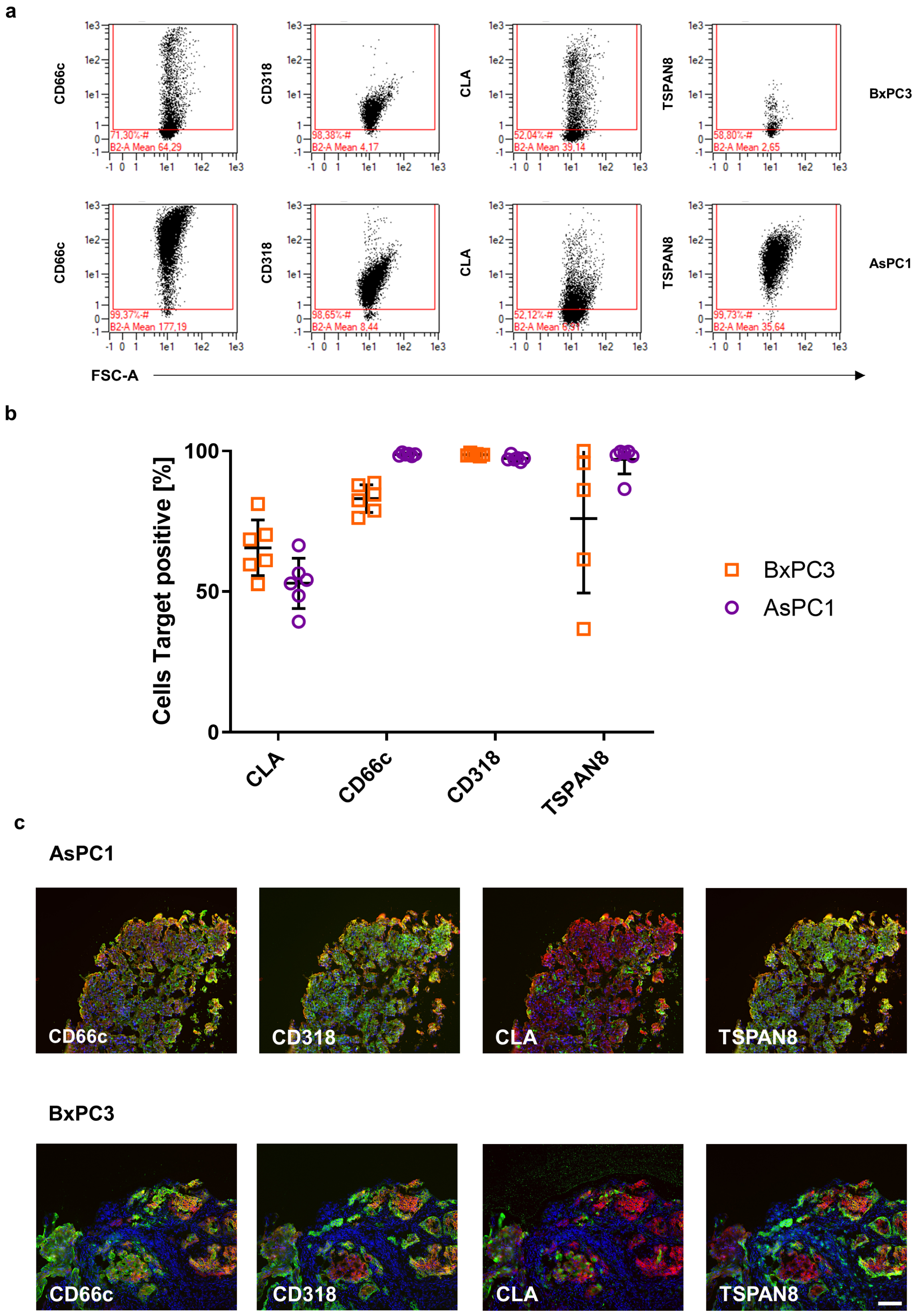
a
CD66c
CD318
CLA
TSPAN8
BxPC3
AsPC1
71,30%-#
B2-A Mean 64,29
98,38%-#
B2-A Mean 4,17
52,04%-#
B2-A Mean 39,14
58,80%-#
B2-A Mean 2,65
99,37%-#
B2-A Mean 177,19
98,65%-#
B2-A Mean 8,44
52,12%-#
99,73%-#
B2-A Mean 35,64
FSC-A
b
Cells Target positive [%]
100
50
0
CLA
CD66c
CD318
TSPAN8
BxPC3
AsPC1
c
AsPC1
CD66c
CD318
CLA
TSPAN8
BxPC3
CD66c
CD318
CLA
TSPAN8

Supplementary Fig. 5: Analysis of target expression upon xenotransplantation of AsPC1 and BxPC3 cells *in vivo*.

The expression of the target candidates was evaluated upon xenotransplantation of AsPC1 and BxPC3 cells *in vivo*. Tumors were grown for 30 days, resected and analyzed for target expression by **(a, b)** flow cytometry (shown are means ± SD, n = 6)**,** and **(c)** immunofluorescence. Scale bar = 100 µm. Images are representative for tumor triplicates. Source data are provided as a Source Data file.

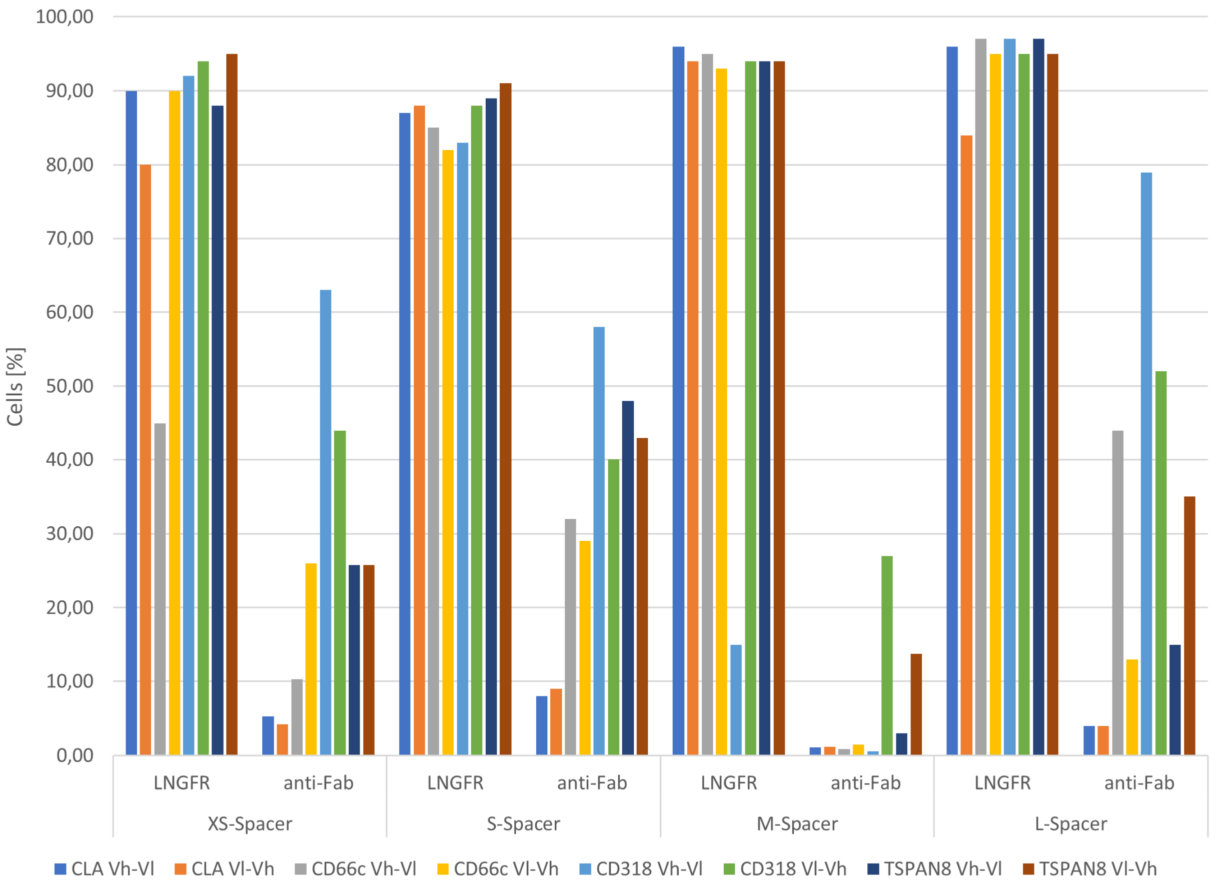

Supplementary Fig. 6: Expression of LNGFR and CAR in HEK293T cells lipofected with the CAR constructs.

Frequency of HEK293T cells which are expressing LNGFR and the respective CAR on the cell surface as measured by flow cytometry. HEK293T cells were lipofected with CAR plasmids to assess the general ability of the CAR cassette in the plasmid to be expressed (as measured by the reporter protein LNGFR) and if the CAR construct can be detected on the cell surface. Data are representative of two independent lipofections. Source data are provided as a Source Data file.

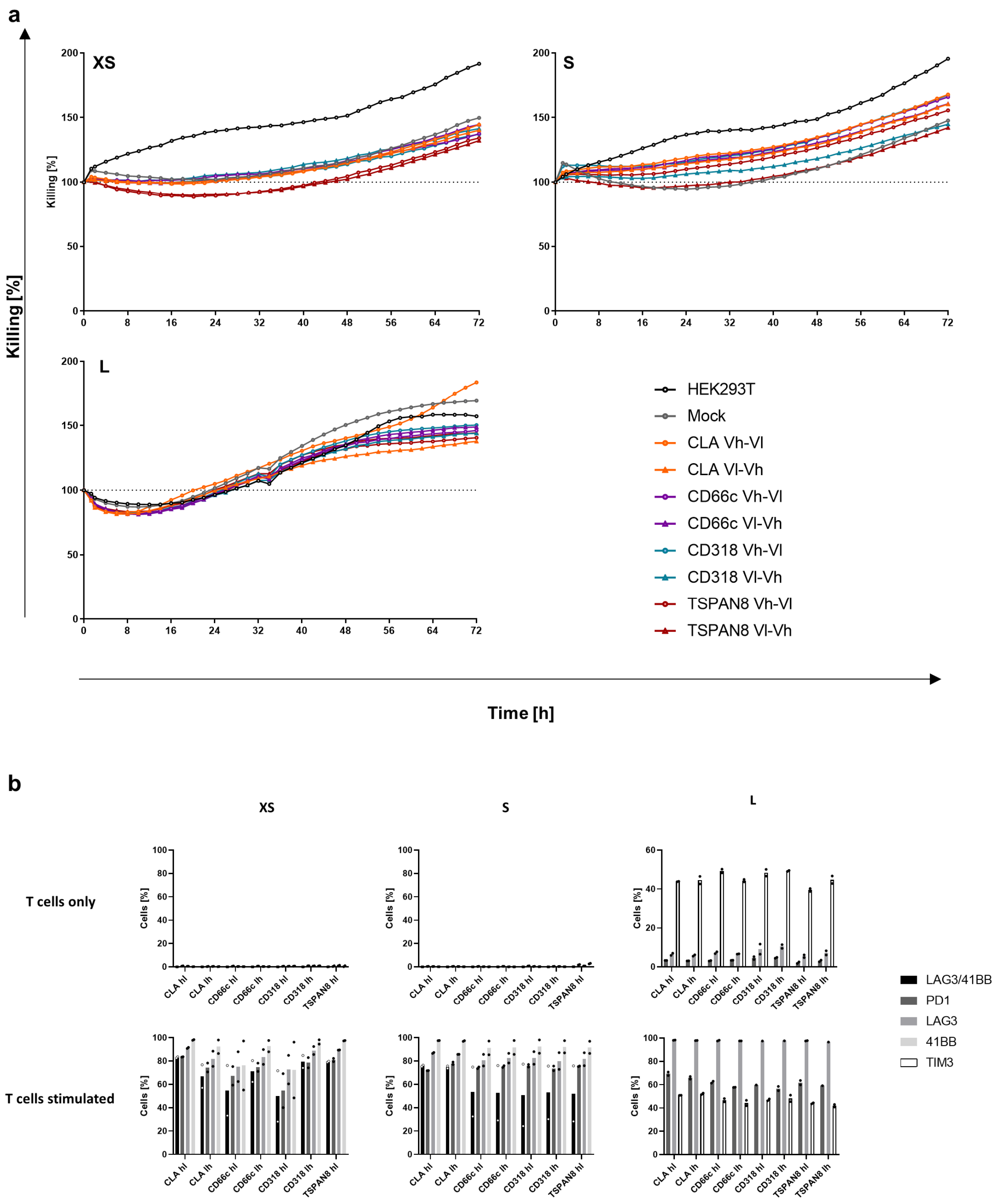

Supplementary Fig. 7: CAR T cells specific for CLA, CD66c, CD318 and TSPAN8 exhibit no cytotoxic effects on target negative cells.

(a) 1x10^5 CAR T cells were inoculated with 5x10^4 HEK293T cells in a 96-well cavity. The amount of Mock T cells was adjusted to the CAR group with the highest amount of total T cells per well. Killing was measured as

percentage difference of green surface area measured automatically with an IncuCyte S3 device. Values represent mean ± s.e.m (n = 2). (**b**) Activation marker expression upon culture without any stimulus or with PMA/Ionomycin stimulation. Shown are the means ± s.d. (n=2). Activation marker expression was measured after 48 h. Source data are provided as a Source Data file.

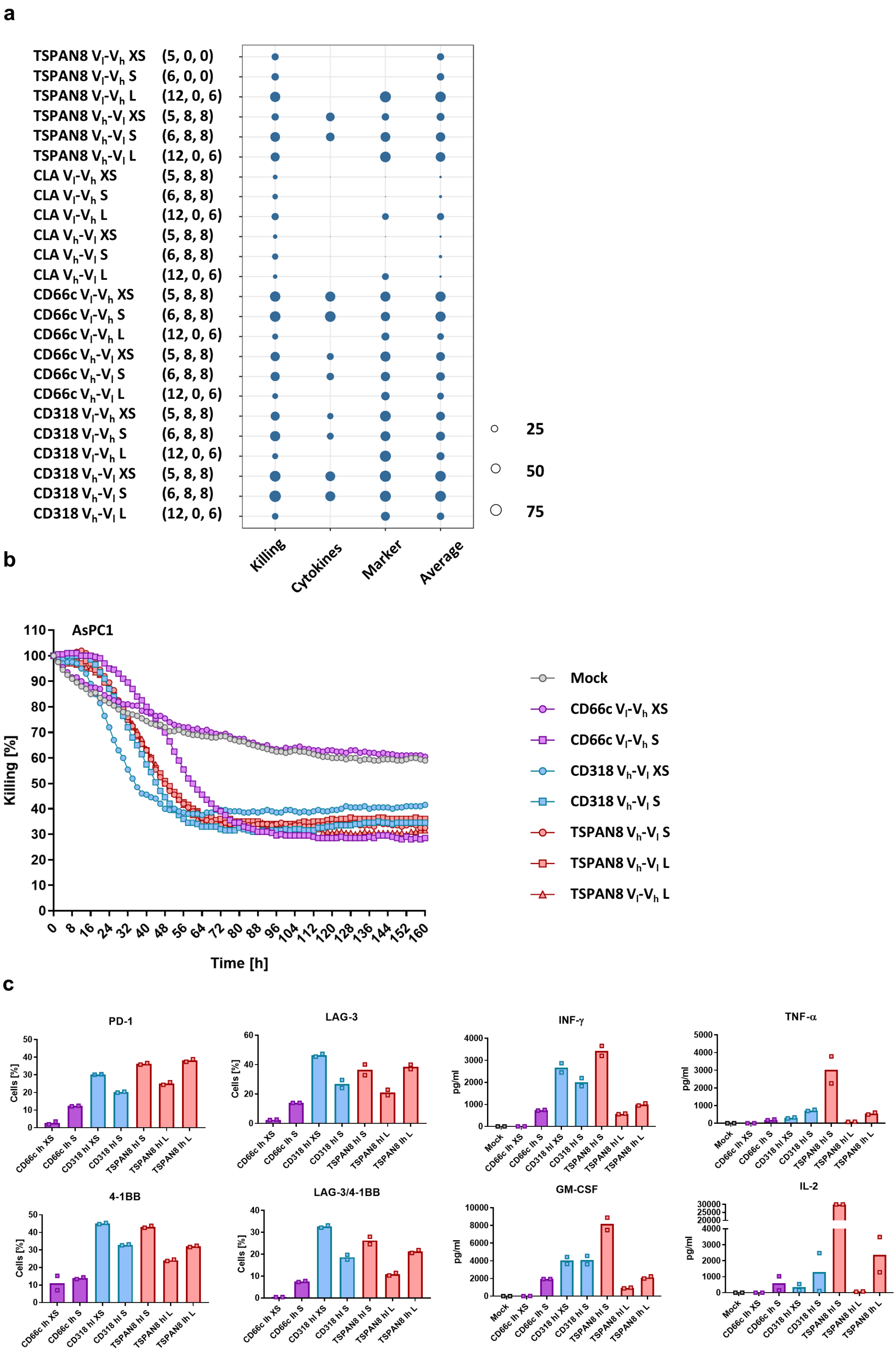
a
TSPAN8 V_l-V_h XS (5, 0, 0)
TSPAN8 V_l-V_h S (6, 0, 0)
TSPAN8 V_l-V_h L (12, 0, 6)
TSPAN8 V_h-V_l XS (5, 8, 8)
TSPAN8 V_h-V_l S (6, 8, 8)
TSPAN8 V_h-V_l L (12, 0, 6)
CLA V_l-V_h XS (5, 8, 8)
CLA V_l-V_h S (6, 8, 8)
CLA V_l-V_h L (12, 0, 6)
CLA V_h-V_l XS (5, 8, 8)
CLA V_h-V_l S (6, 8, 8)
CLA V_h-V_l L (12, 0, 6)
CD66c V_l-V_h XS (5, 8, 8)
CD66c V_l-V_h S (6, 8, 8)
CD66c V_l-V_h L (12, 0, 6)
CD66c V_h-V_l XS (5, 8, 8)
CD66c V_h-V_l S (6, 8, 8)
CD66c V_h-V_l L (12, 0, 6)
CD318 V_l-V_h XS (5, 8, 8)
CD318 V_l-V_h S (6, 8, 8)
CD318 V_l-V_h L (12, 0, 6)
CD318 V_h-V_l XS (5, 8, 8)
CD318 V_h-V_l S (6, 8, 8)
CD318 V_h-V_l L (12, 0, 6)
25
50
75
Killing
Cytokines
Marker
Average
b
AsPC1
Killing [%]
Time [h]
Mock
CD66c V_l-V_h XS
CD66c V_l-V_h S
CD318 V_h-V_l XS
CD318 V_h-V_l S
TSPAN8 V_h-V_l S
TSPAN8 V_h-V_l L
TSPAN8 V_l-V_h L
c
PD-1
LAG-3
INF-γ
TNF-α
4-1BB
LAG-3/4-1BB
GM-CSF
IL-2
Cells [%]
pg/ml

Supplementary Fig. 8: Evaluation of CAR T cell functionality *in vitro*.

(**a**) Results of the initial screening assays. Average relative target cell killing, cytokine release, marker upregulation and overall performance (displayed as circle size, x axis) for the respective CAR constructs (y axis). The number of replicates is indicated in parentheses behind the construct name. (First value = n of killing assays, second value = n of cytokine release assays, third value = n of marker upregulation measurements). (**b**) Representative result for the kinetics of AsPC1 target cell killing by the best performing CAR constructs. (**c**) Representative results showing cytokine release and activation marker expression patterns upon co-culture with AsPC1 target cells. Shown are the means ± s.e.m (n=2). Activation marker expression was measured at end point of cytotoxicity assay, cytokine release patterns were measured after 48 h. Source data are provided as a Source Data file.

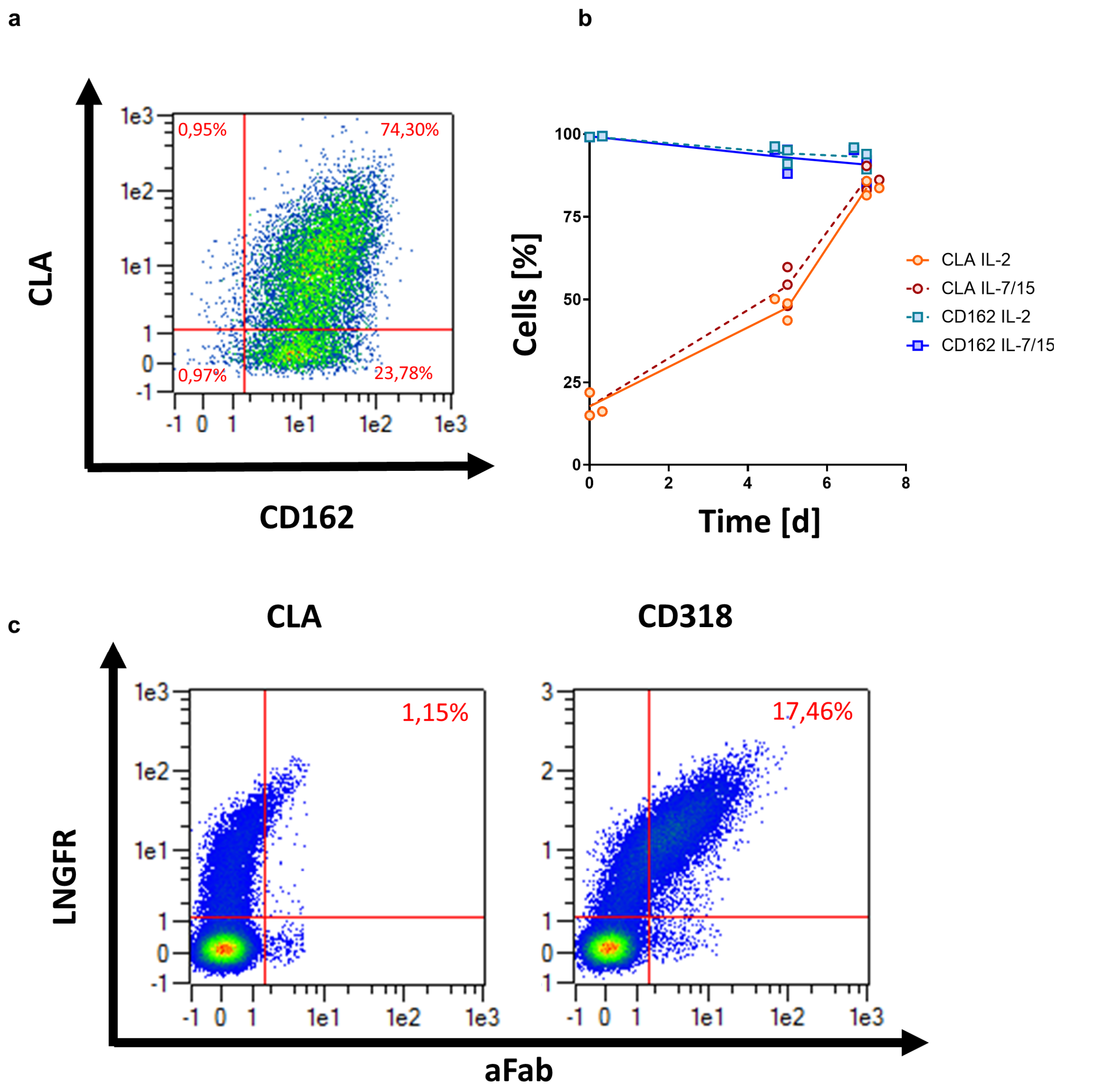

Supplementary Figure 9: CLA is a expressed on T cells and upregulated upon activation.

(a) Expression of CLA and its protein backbone CD162 on T cells upon activation and expansion for 7 days. **(b)** Kinetic of CLA and CD162 expression on T cells followed over 7 days. Depicted are the single measurements of three donors. Isolated T cells were activated with T Cell TransAct™, human and cultivated in medium either supplemented with 100 IU/ml IL-2 or 12.5 ng/mL of recombinant human interleukin IL-7 and 12.5 ng/mL of recombinant human IL-15. **(c)** Although the CAR cassette is functional based on LNGFR expression, the a anti-CLA CAR is expressed only at low levels in LNGFR^{high} CAR T cells as compared to a CD318 specific CAR. Source data are provided as a Source Data file.

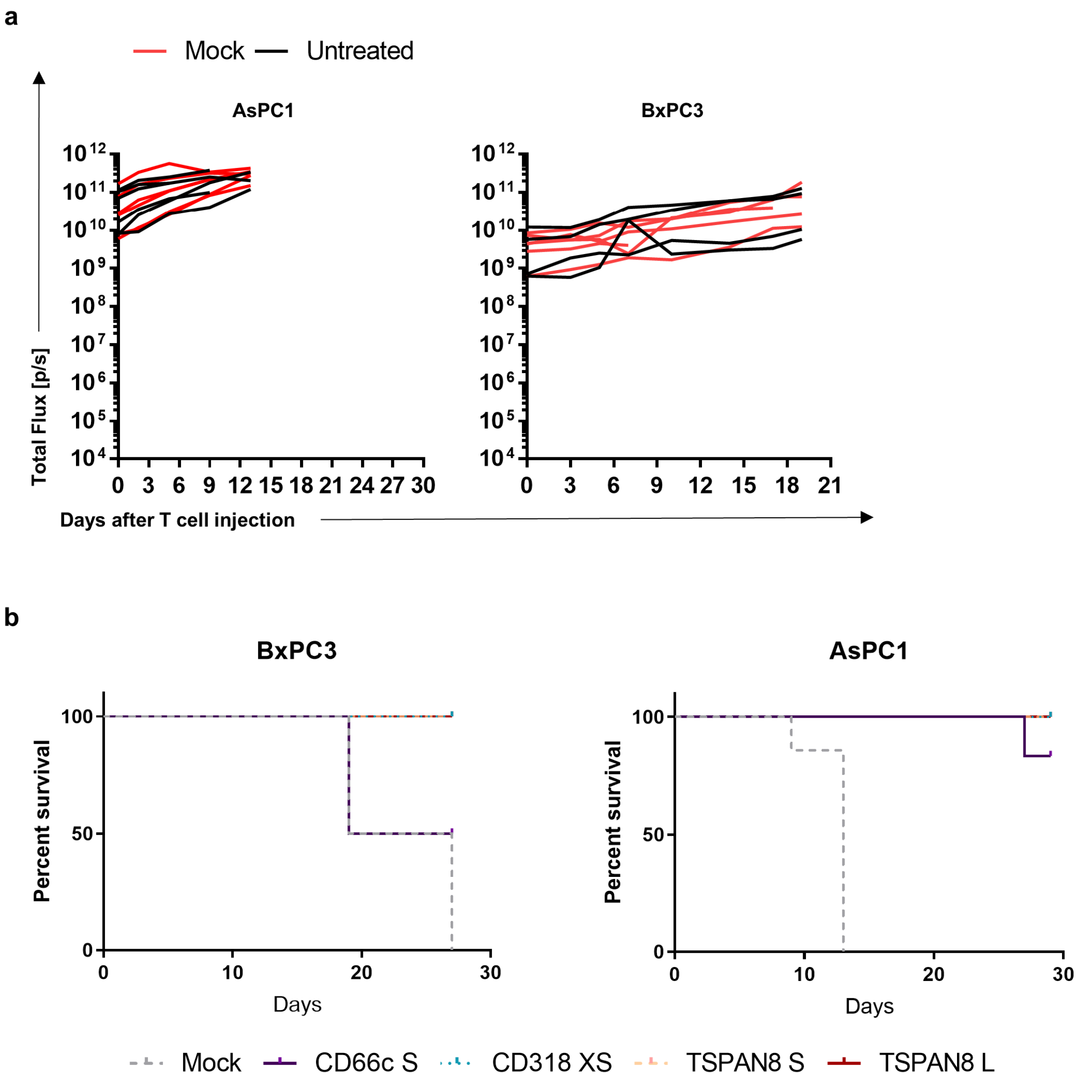

Supplementary Fig. 10: Mock T cells have no effect on tumor outgrowth in both *in vivo* models.

(a) Tumor burden of mice treated either with Mock T cells or untreated. Left: Mice injected with $1x10^6$ AsPC1 cells (Mock: n = 6, Untreated: n = 5). Right: Mice injected with $1x10^6$ BxPC3 cells (Mock: n = 6, Untreated n = 4). **(b)** Kaplan-Meier plots of mice treated with T cells. (For AsPC1: Mock: n = 6, CD66c S: n = 6, CD318 XS: n = 6, TSPAN8 S: n = 4, TSPAN8 L: n = 5; for BxPC3: Mock: n = 4, CD66c S: n = 4, CD318 XS: n = 4, TSPAN8 S: n = 4, TSPAN8 L: n = 4; animals taken out for interim analysis were not included). Source data are provided as a Source Data file.

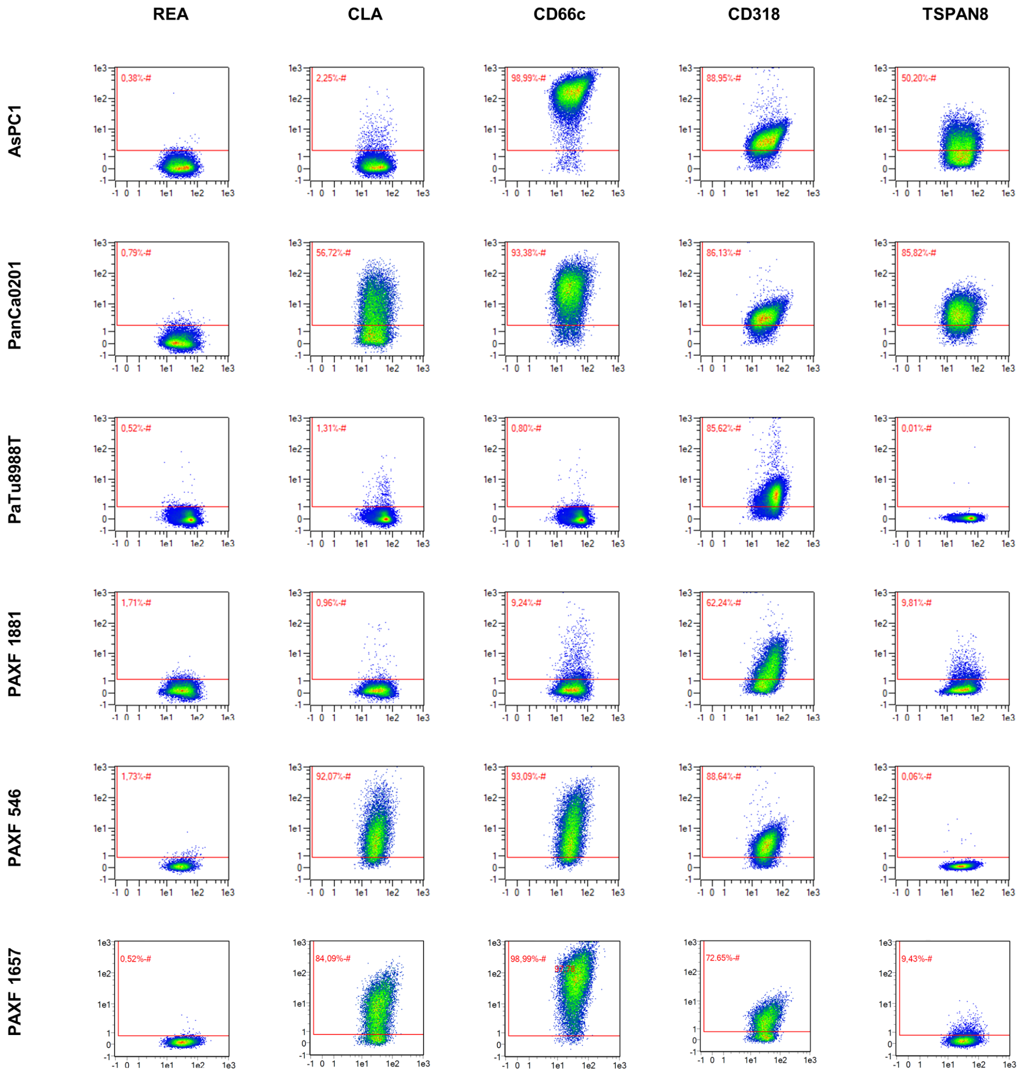

Supplementary Fig. 11: Analysis of target expression on primary cells, cell lines, and PDX models.

The expression of the target candidates was measured by flow cytometry on primary cells (PanCa0201) derived from a human PDAC and passaged less than 10 times, cell lines (AsPC1 and PaTu8988T), and freshly dissociated PDX models (PAXF1881/546/1657). REA = isotype control staining.

CD318

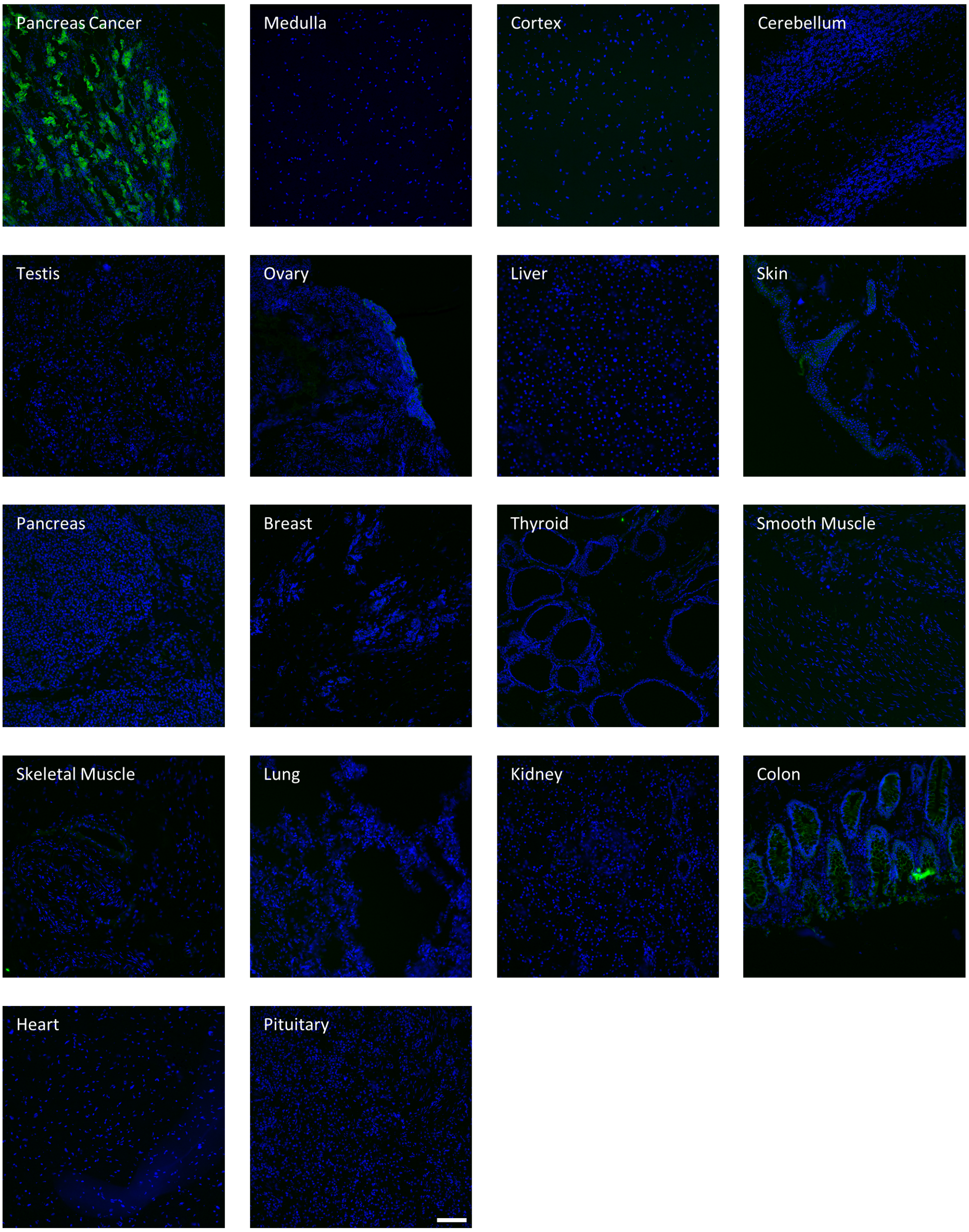

Pancreas Cancer
Medulla
Cortex
Cerebellum
Testis
Ovary
Liver
Skin
Pancreas
Breast
Thyroid
Smooth Muscle
Skeletal Muscle
Lung
Kidney
Colon
Heart
Pituitary

Supplementary Fig. 12: CD318 expression on healthy tissues assessed by cyclic immunofluorescence imaging. Representative cyclic immune fluorescence images of several healthy tissues stained with a CD318-PE conjugate. Scale bar = 100 µm. Images are representative for at least two regions of interest from one tissue. Regions of interest were chosen based on manual DAPI and Cytokeratin prestaining and in dependency to the respective tissue size.

TSPAN8

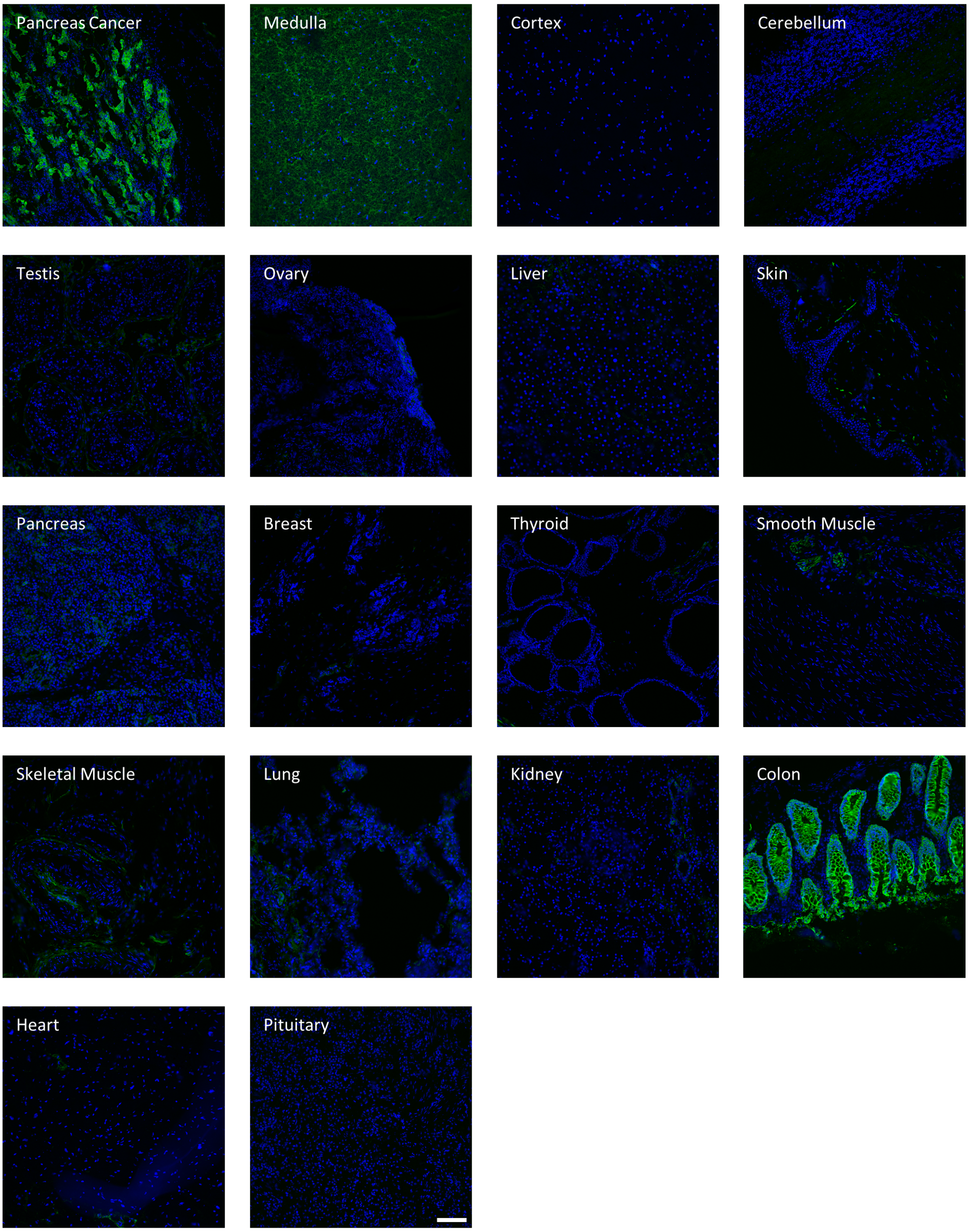
Pancreas Cancer
Medulla
Cortex
Cerebellum
Testis
Ovary
Liver
Skin
Pancreas
Breast
Thyroid
Smooth Muscle
Skeletal Muscle
Lung
Kidney
Colon
Heart
Pituitary

Supplementary Fig. 13: TSPAN8 expression on healthy tissues assessed by cyclic immunofluorescence imaging.

Representative cyclic immune fluorescence images of several healthy tissues stained with a TSPAN8-PE conjugate. Scale bar = 100 µm. Images are representative for at least two regions of interest from one tissue. Regions of interest were chosen based on manual DAPI and Cytokeratin prestaining and in dependency to the respective tissue size.

CD66c

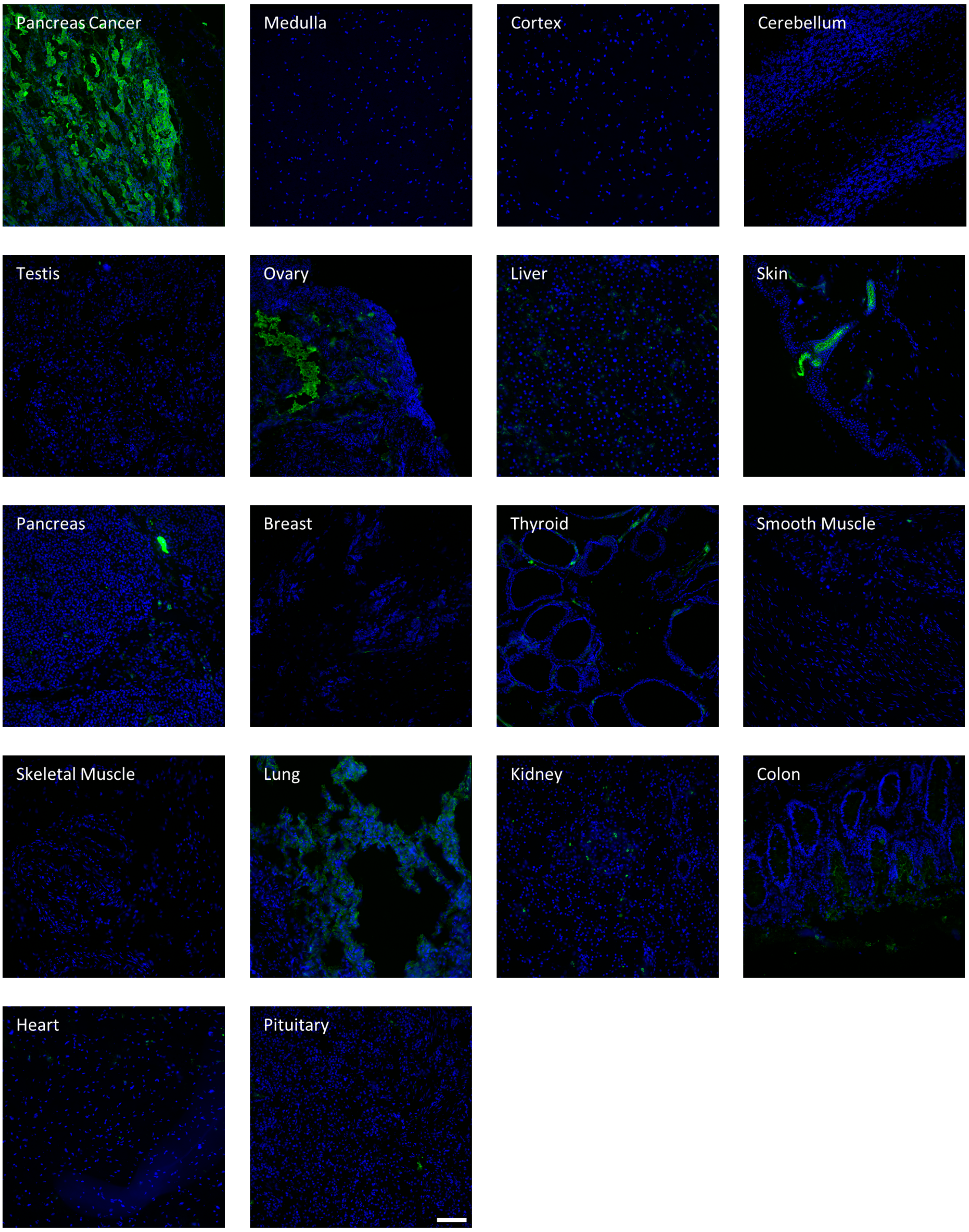
Pancreas Cancer
Medulla
Cortex
Cerebellum
Testis
Ovary
Liver
Skin
Pancreas
Breast
Thyroid
Smooth Muscle
Skeletal Muscle
Lung
Kidney
Colon
Heart
Pituitary

Supplementary Fig. 14: CD66c expression on healthy tissues assessed by cyclic immunofluorescence imaging. Representative cyclic immune fluorescence images of several healthy tissues stained with a CD66c-PE conjugate. Scale bar = 100 µm. Images are representative for at least two regions of interest from one tissue. Regions of interest were chosen based on manual DAPI and Cytokeratin prestaining and in dependency to the respective tissue size.

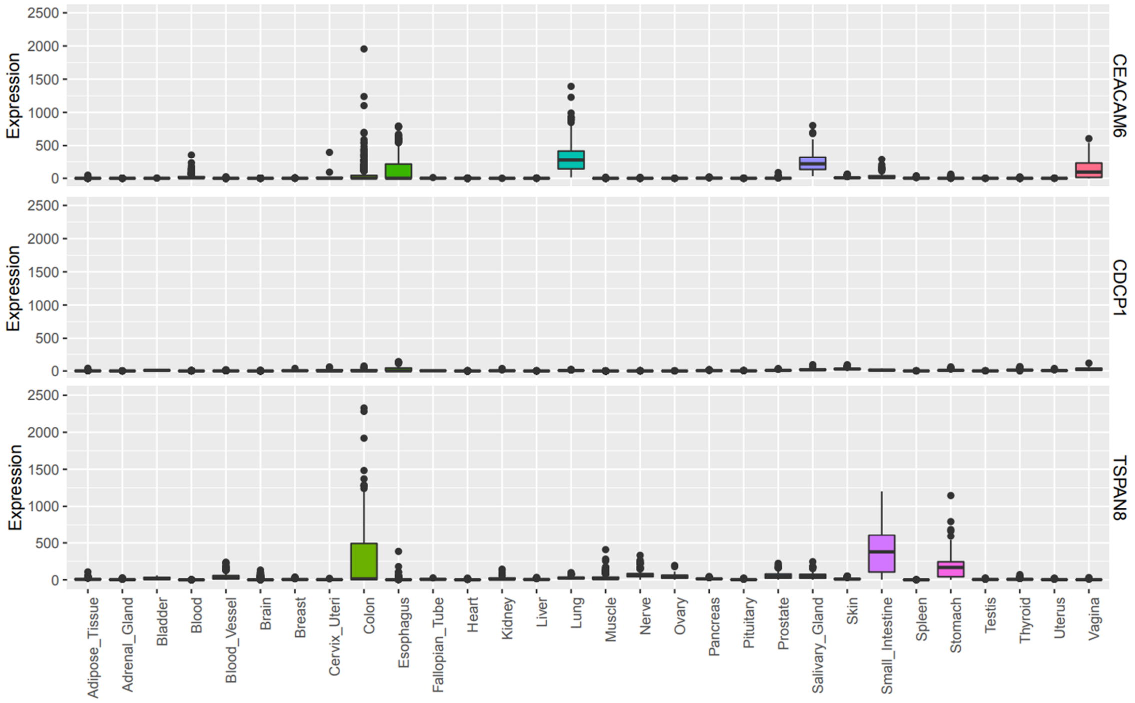

Supplementary Fig. 15: Overview of healthy tissue expression for determining possible target combinations. RNA expression of target candidates derived from GTEx (v7) indicating which combinations would be suitable for combinatorial CAR approaches. The lower and upper hinges in the box-and-whisker plots correspond to the first and third quartile (25th and 75th percentiles). The bar in the box depicts the median. The upper whisker spans from hinge to the highest value with a distance of not more than 1.5x of the inter-quartile range. The lower whisker spans from hinge to the lowest value of not more than 1.5x inter-quartile range. Data beyond the whiskers are plotted individually. As shown, no significant overlap could be observed among healthy tissues. CEACAM6 = CD66c, CDCP1 = CD318. Number of samples as from GTEx (v7).
