# Supplementary material for: Identification of CD318, TSPAN8 and CD66c as target candidates for CAR T cell based immunotherapy of pancreatic adenocarcinoma
Source: Nat Commun. 2021 Mar 5;12:1453. doi: 10.1038/s41467-021-21774-4 (PMC7935963; doi:10.1038/s41467-021-21774-4)
Supplement: Supplementary file 3 — Descriptions of Additional Supplementary Files [file 41467_2021_21774_MOESM3_ESM.pdf]

Descriptions of Additional Supplementary Files

Supplementary Data 1

Description: PDX mutational landscape

Supplementary Data 2

Description: Bioinformatics based ranking of target candidates

Supplementary Data 3

Description: Bioinformatics based ranking of target candidates with essential tissues

Supplementary Data 4

Description: Bioinformatics based ranking of target candidates with non-essential tissues

Supplementary Data 5

Description: Antibodies used in this study

Supplementary Data 6

Description: PDAC target expression on primary and metastatic tissue - TCGA

Supplementary 7 Data

Description: Raw-Data of the *in vitro* assays performed with all L, S and XS spacer CAR T cells

Supplementary Data 8

Description: Raw-Data of the *in vitro* assays performed with the most efficient CAR T cells for selection of the *in vivo* candidates

Supplementary Movie 1

Description: Mock killing of BxPC3 cells

Supplementary Movie 2

Description: aCD66c CAR T cell killing of BxPC3
