# Supplementary material for: Identification of CD318, TSPAN8 and CD66c as target candidates for CAR T cell based immunotherapy of pancreatic adenocarcinoma
Source: Nat Commun. 2021 Mar 5;12:1453. doi: 10.1038/s41467-021-21774-4 (PMC7935963; doi:10.1038/s41467-021-21774-4)
Supplement: Supplementary file 14 — Reporting summary [file 41467_2021_21774_MOESM14_ESM.pdf]

nature research

Corresponding author(s): Olaf Hardt

Last updated by author(s): Jan 19, 2021

Reporting Summary

Nature Research wishes to improve the reproducibility of the work that we publish. This form provides structure for consistency and transparency in reporting. For further information on Nature Research policies, see our Editorial Policies and the Editorial Policy Checklist.

Statistics

For all statistical analyses, confirm that the following items are present in the figure legend, table legend, main text, or Methods section.

n/a	Confirmed	
☐	☒	The exact sample size (n) for each experimental group/condition, given as a discrete number and unit of measurement
☐	☒	A statement on whether measurements were taken from distinct samples or whether the same sample was measured repeatedly
☐	☒	The statistical test(s) used AND whether they are one- or two-sided<br>*Only common tests should be described solely by name; describe more complex techniques in the Methods section.*
☒	☐	A description of all covariates tested
☐	☒	A description of any assumptions or corrections, such as tests of normality and adjustment for multiple comparisons
☐	☒	A full description of the statistical parameters including central tendency (e.g. means) or other basic estimates (e.g. regression coefficient) AND variation (e.g. standard deviation) or associated estimates of uncertainty (e.g. confidence intervals)
☐	☒	For null hypothesis testing, the test statistic (e.g. F, t, r) with confidence intervals, effect sizes, degrees of freedom and P value noted<br>*Give P values as exact values whenever suitable.*
☒	☐	For Bayesian analysis, information on the choice of priors and Markov chain Monte Carlo settings
☒	☐	For hierarchical and complex designs, identification of the appropriate level for tests and full reporting of outcomes
☒	☐	Estimates of effect sizes (e.g. Cohen's d, Pearson's r), indicating how they were calculated

Our web collection on statistics for biologists contains articles on many of the points above.

Software and code

Policy information about availability of computer code

Data collection	MACS Quantify v2.13.0, MACSima Prototype, Living Image Software (v4.4), IncuCyte S3 (v2017A, v2018C and v2019A), FlowJow v10.7.1
Data analysis	GraphPad Prism V7, Living Image 4.4, R v3.4 with the packages reshape2, ggplot2 and viridis, MeV v4.9.0, Microsoft Excel for Windows 2016, ImageJ 1.49v, CellProfiler v2.2.0, MeV 4.9.0

For manuscripts utilizing custom algorithms or software that are central to the research but not yet described in published literature, software must be made available to editors and reviewers. We strongly encourage code deposition in a community repository (e.g. GitHub). See the Nature Research guidelines for submitting code & software for further information.

Data

Policy information about availability of data

All manuscripts must include a data availability statement. This statement should provide the following information, where applicable:

- Accession codes, unique identifiers, or web links for publicly available datasets
- A list of figures that have associated raw data
- A description of any restrictions on data availability

Data are available from the corresponding author upon reasonable request. Publicly available data were retrieved from: Human Protein Atlas (26) (v15, https://www.proteinatlas.org/), ProteomicsDB (27) (https://www.proteomicsdb.org/), human proteome map (28) (http://www.humanproteomemap.org/), Genotype-Tissue Expression (GTEx, https://gtexportal.org/), genevestigator (73) and the TCGA Research Network (https://www.cancer.gov/tcga).

April 2020

Field-specific reporting

Please select the one below that is the best fit for your research. If you are not sure, read the appropriate sections before making your selection.

- [x] Life sciences
- [ ] Behavioural & social sciences
- [ ] Ecological, evolutionary & environmental sciences

For a reference copy of the document with all sections, see nature.com/documents/nr-reporting-summary-flat.pdf

Life sciences study design

All studies must disclose on these points even when the disclosure is negative.

Sample size

No statistical methods were used to determine sample sizes for in vitro assays. Our experience showed that absolute differences between donors occur in the setting of CAR construct evaluation, but the relative differences stay the same. Thus for initially narrowig down the field of CAR candidates it was sufficient to repeat an experiment at least twice, while the final constructs were evaluated in biological triplicates plus the previous pre-selection assays. All results were confirming each other. Final constructs were also tested in vivo and on xenografts, confiming their robustness. Sample sizes for in vivo assays were were calculated using a statistical software and estimated based on prelimnary experiments and the invetsigators experience with trying to achieve a minimum of n=4 mice per group.

Data exclusions

No data were excluded during this study.

Replication

During the course of this study we replicated each experiment at least once, with no contradictory outcomes. In addition, we chose to investigate if same outcomes could be confirmed with different techniques or on differrent samples increasing the robustness of our findings (verifying flow cytometry with microscopy, verifying PDX findings on primary tumors, choosing different models to challange the same CAR, etc.)

Randomization

Animals were assigned to treatment groups using randomization based on bioluminescence and tumor size, to ensure equal distribution of bioluminescence signal and tumor size between the groups.
For histological and immunofluorescence microscopy, all tissues were cut at random sites and stained and allocated in random order. Patient samples were chosen by surgeons of the Universitätsklinik Göttingen, who decided based on teratment guidlines, which patient should undergo surgery and samples were forwared to the investigators or supplied by the commercial source Proteogenex, which chose the patient samples. T cells are derived from random donors.

Blinding

Except for the caliper measurement of the tumor size, all other data analyses were based on objectively measurable data. Caliper measurements were conducted during the whole study by the same investigator. The investigator was blinded before assessing tumor size. For other experiments blindig was not strictly required as outcomes were based on objective measures. Experiments were designed to prepare and measure severeal samples simultaneously in a uniform way. Apprpriate controls were alsways included.

Reporting for specific materials, systems and methods

We require information from authors about some types of materials, experimental systems and methods used in many studies. Here, indicate whether each material, system or method listed is relevant to your study. If you are not sure if a list item applies to your research, read the appropriate section before selecting a response.

Materials & experimental systems

n/a	Involved in the study	
	x	Antibodies
	x	Eukaryotic cell lines
x		Palaeontology and archaeology
	x	Animals and other organisms
	x	Human research participants
x		Clinical data
x		Dual use research of concern

Methods

n/a	Involved in the study	
x		ChIP-seq
	x	Flow cytometry
x		MRI-based neuroimaging

Antibodies

Antibodies used

Antigen Clone Isotype Titer Order Nr. Vendor
DAPI x x 1/10 130-111-570 Miltenyi Biotec
CD3 BW264/56 mouse IgG2a 1/11 130-113-129 Miltenyi Biotec
CD4 vit4 mouse IgG2aκ 1/11 130-113-214 Miltenyi Biotec
CD8 BW135/80 mouse IgG2a 1/11 130-113-158 Miltenyi Biotec
CD9 SN4 C3-3A2 mouse IgG1κ 1/7,5 130-123-761 Miltenyi Biotec
CD11c MJ4-27G12 mouse IgG2b 1/2,5 130-113-580 Miltenyi Biotec
CD14 TÜK4 mouse IgG2a 1/2,5 130-113-147 Miltenyi Biotec

CD15 VIMC6 mouse IgM 1/5 130-113-485 Miltenyi Biotec
CD18 TS1/18 mouse IgG1κ 1/11 130-101-241 Miltenyi Biotec
CD19 LT19 mouse IgG1 1/2,5 130-113-169 Miltenyi Biotec
CD20 REA780 recombinant human IgG1 1/5 130-111-338 Miltenyi Biotec
CD23 M-L23.4 mouse IgG1 1/5 130-123-716 Miltenyi Biotec
CD24 32D12 mouse IgG1κ 1/5 130-095-952 Miltenyi Biotec
CD27 REA499 recombinant human IgG1 1/20 130-113-640 Miltenyi Biotec
CD28 15e8 mouse IgG1 1/2,5 130-092-921 Miltenyi Biotec
CD29 TS2/16 mouse IgG1κ 1/11 130-101-273 Miltenyi Biotec
CD31 AC128 mouse IgG1κ 1/20 130-118-965 Miltenyi Biotec
CD34 AC136 mouse IgG2aκ 1/5 130-113-179 Miltenyi Biotec
CD38 IB6 mouse IgG2a 1/15 130-113-427 Miltenyi Biotec
CD40 HB14 mouse IgG1κ 1/40 130-123-714 Miltenyi Biotec
CD44 DB105 mouse IgG1 1/60 130-113-335 Miltenyi Biotec
CD45RO UCHL1 mouse IgG2aκ 1/11 130-095-457 Miltenyi Biotec
CD45RA T6D11 mouse IgG2b 1/35 130-113-356 Miltenyi Biotec
CD45 5B1 mouse IgG2a 1/80 130-113-118 Miltenyi Biotec
CD46 REA312 recombinant human IgG1 1/15 130-104-508 Miltenyi Biotec
CD47 REA220 recombinant human IgG1 1/15 130-123-754 Miltenyi Biotec
CD49B REA188 recombinant human IgG1 1/2,5 130-123-749 Miltenyi Biotec
CD49c REA360 recombinant human IgG1 1/7,5 130-118-544 Miltenyi Biotec
CD49e NKI-SAM1 mouse IgG2bκ 1/11 130-097-225 Miltenyi Biotec
CD49f GoH3 rat IgG2aκ 1/20 130-097-245 Miltenyi Biotec
CD51 REA181 recombinant human IgG1 1/2,5 130-100-556 Miltenyi Biotec
CD54 REA266 recombinant human IgG1 1/60 130-120-711 Miltenyi Biotec
CD55 JS11 mouse IgG1κ 1/7,5 130-101-764 Miltenyi Biotec
CD56 REA196 recombinant human IgG1 1,25 130-113-312 Miltenyi Biotec
CD58 TS2/9 mouse IgG1κ 1/5 130-101-193 Miltenyi Biotec
CD59 REA496 recombinant human IgG1 1/65 130-120-048 Miltenyi Biotec
CD63 REA563 recombinant human IgG1 1/60 130-108-894 Miltenyi Biotec
CD66acde REA428 recombinant human IgG1 1/40 130-125-211 Miltenyi Biotec
CD66c REA414 recombinant human IgG1 1/45 130-123-270 Miltenyi Biotec
CD68 Y1/82A mouse IgG2bκ 1/7,5 130-118-348 Miltenyi Biotec
CD71 AC102 mouse IgG2aκ 1/11 130-091-728 Miltenyi Biotec
CD73 AD2 mouse IgG1κ 1/5 130-120-066 Miltenyi Biotec
CD80 2D10 mouse IgG1κ 1/20 130-117-683 Miltenyi Biotec
CD82 REA221 recombinant human IgG1 1/11 130-101-306 Miltenyi Biotec
TRA-1-85 REA476 recombinant human IgG1 1/11 130-107-102 Miltenyi Biotec
CD86 FM95 mouse IgG1κ 1/1 130-113-572 Miltenyi Biotec
CD90 DG3 mouse IgG1κ 1/5 130-117-388 Miltenyi Biotec
CD94 REA113 recombinant human IgG1 1/2,5 130-098-974 Miltenyi Biotec
CD95 DX2 mouse IgG1κ 1/5 130-123-706 Miltenyi Biotec
CD97 VIM3b mouse IgG1κ 1/11 130-097-102 Miltenyi Biotec
CD98 REA387 recombinant human IgG1 1/20 130-120-051 Miltenyi Biotec
CD104 REA236 recombinant human IgG1 1/2,5 130-123-756 Miltenyi Biotec
CD105 43A4E1 mouse IgG1κ 1/7,5 130-117-696 Miltenyi Biotec
CD107a H4A3 mouse IgG1κ 1/20 130-119-872 Miltenyi Biotec
CD117 A3C6E2 mouse IgG1κ 1/2,5 130-113-544 Miltenyi Biotec
CD119 REA161 recombinant human IgG1 1/11 130-125-851 Miltenyi Biotec
CD133 AC133 mouse IgG1κ 1/5 130-113-108 Miltenyi Biotec
CD134 ACT35 mouse IgG1κ 1/2,5 130-116-488 Miltenyi Biotec
CD138 44F9 mouse IgG1 1/2,5 130-119-840 Miltenyi Biotec
CD141 AD5-14H12 mouse IgG1 1/2,5 130-113-318 Miltenyi Biotec
CD142 HTF-1 mouse IgG1κ 1/2,5 130-098-742 Miltenyi Biotec
CD146 541-10B2 mouse IgG1κ 1/11 130-123-711 Miltenyi Biotec
CD147 REA282 recombinant human IgG1 1/11 130-123-764 Miltenyi Biotec
CD151 REA265 recombinant human IgG1 1/11 130-103-662 Miltenyi Biotec
CD152 BNI3 mouse IgG2aκ 1/1 130-118-357 Miltenyi Biotec
CD155 PV404.19 mouse IgG1 1/2,5 130-105-846 Miltenyi Biotec
CD156c REA309 recombinant human IgG1 1/5 130-104-407 Miltenyi Biotec
CD162 REA319 recombinant human IgG1 1/20 130-104-707 Miltenyi Biotec
CD163 GHI/61.1 mouse IgG1κ 1/20 130-123-249 Miltenyi Biotec
CD166 REA442 recombinant human IgG1 1/11 130-118-349 Miltenyi Biotec
CD171 REA163 recombinant human IgG1 1/2,5 130-100-691 Miltenyi Biotec
CD178 NOK-1 mouse IgG1κ 1/2,5 130-118-353 Miltenyi Biotec
CD183 REA232 recombinant human IgG1 1/15 130-120-452 Miltenyi Biotec
CD184 12G5 mouse IgG2aκ 1/5 130-117-690 Miltenyi Biotec
CD195 REA245 recombinant human IgG1 1/5 130-117-356 Miltenyi Biotec

CD204 REA460 recombinant human IgG1 1/11 130-119-580 Miltenyi Biotec
CD206 DCN228 mouse IgG1κ 1/7,5 130-124-233 Miltenyi Biotec
CD223 REA351 recombinant human IgG1 1/5 130-120-470 Miltenyi Biotec
CD227 REA448 recombinant human IgG1 1/11 130-120-056 Miltenyi Biotec
CD230 REA203 recombinant human IgG1 1/11 130-101-304 Miltenyi Biotec
CD239 REA276 recombinant human IgG1 1/11 130-103-842 Miltenyi Biotec
CD240DCE REA327 recombinant human IgG1 1/5 130-104-819 Miltenyi Biotec
CD266 REA1216 recombinant human IgG1 1/11 130-123-395 Miltenyi Biotec
CD273 MIH18 mouse IgG1κ 1/5 130-098-530 Miltenyi Biotec
CD274 29E.2A3 mouse IgG2bκ 1/20 329705 BioLegend
CD276 FM276 mouse IgG2bκ 1/30 130-120-712 Miltenyi Biotec
CD278 REA192 recombinant human IgG1 1/5 130-120-069 Miltenyi Biotec
CD279 PD1.3.1.3 mouse IgG2b 1/11 130-117-384 Miltenyi Biotec
CD298 REA217 recombinant human IgG1 1/11 130-101-290 Miltenyi Biotec
CD309 ES8-20E6 mouse IgG1κ 1/2,5 130-120-480 Miltenyi Biotec
CD317 REA202 recombinant human IgG1 1/7,5 130-101-707 Miltenyi Biotec
CD318 REA194 recombinant human IgG1 1/2,5 130-101-215 Miltenyi Biotec
CD324 REA811 recombinant human IgG1 1/5 130-111-839 Miltenyi Biotec
CD326 HEA-125 mouse IgG1κ 1/5 130-113-264 Miltenyi Biotec
GITR/CD357 DT5D3 mouse IgG1κ 1/2,5 130-121-331 Miltenyi Biotec
CD366 REA635 recombinant human IgG1 1/11 130-119-785 Miltenyi Biotec
CX3CR1 REA385 recombinant human IgG1 1/50 130-122-912 Miltenyi Biotec
Oct-4 REA338 recombinant human IgG1 1/5 130-117-709 Miltenyi Biotec
TSPAN-8 REA443 recombinant human IgG1 1/11 130-117-391 Miltenyi Biotec
SSEA-1 REA321 recombinant human IgG1 1/2,5 130-117-689 Miltenyi Biotec
Anti SSEA-4 REA101 recombinant human IgG1 1/5 130-122-914 Miltenyi Biotec
VEGFR-1 REA569 recombinant human IgG1 1/11 130-124-438 Miltenyi Biotec
HLA-DR AC122 mouse IgG2aκ 1/70 130-113-402 Miltenyi Biotec
FoxP3 3G3 mouse IgG1κ 1/100 MABF463 Sigma-Aldrich
Ki67 REA183 recombinant human IgG1 1/2,5 130-120-417 Miltenyi Biotec
Vimentin REA409 recombinant human IgG1 1/2,5 130-116-508 Miltenyi Biotec
Podoplanin REA446 recombinant human IgG1 1/7,5 130-117-687 Miltenyi Biotec
IgD IgD26 mouse IgG1κ 1/11 130-094-539 Miltenyi Biotec
IgA IS11-8E10 mouse IgG1κ 1/20 130-113-476 Miltenyi Biotec
IgG IS11-3B2.2.3 mouse IgG1κ 1/5 130-119-878 Miltenyi Biotec
HLA-A2 A28 REA142 recombinant human IgG1 1/40 130-099-536 Miltenyi Biotec
HLA-ABC REA230 recombinant human IgG1 1/11 130-120-055 Miltenyi Biotec
Cytokeratin REA831 recombinant human IgG1 1/50 130-112-744 Miltenyi Biotec
Rat IgM – isotype control antibodies ES26-13D3.4 rat IgMκ 1/11 130-102-672 Miltenyi Biotec
Mouse IgG1 – isotype control antibodies IS5-21F5 mouse IgG1κ 1/11 130-113-200, Miltenyi Biotec
REA Control antibodies REA293 recombinant human IgG1 1/11 130-113-450 Miltenyi Biotec
HLA-DQ REA303 recombinant human IgG1 1/80 130-104-496 Miltenyi Biotec
Anti-CLA HECA-452 rat IgMκ 1/7,5 130-123-707 Miltenyi Biotec
Antigen Clone Isotype Titer Order Nr. Vendor
CD49c REA360 recombinant human IgG1 1/50 130-118-544 Miltenyi Biotec
CD66c REA414 recombinant human IgG1 1/50 130-123-270 Miltenyi Biotec
CD73 REA778 recombinant human IgG1 1/50 130-111-331 Miltenyi Biotec
CD104 REA236 recombinant human IgG1 1/50 130-123-756 Miltenyi Biotec
CD142 REA949 recombinant human IgG1 1/50 130-115-684 Miltenyi Biotec
CD318 REA194 recombinant human IgG1 1/50 130-101-215 Miltenyi Biotec
TSPAN8 REA443 recombinant human IgG1 1/50 130-117-391 Miltenyi Biotec
CLA HECA-452 rat IgMκ 1/11 130-123-707 Miltenyi Biotec
CLA REA1101 recombinant human IgG1 1/50 130-119-043 Miltenyi Biotec
EpCAM REA764 recombinant human IgG1 1/50 130-110-998 Miltenyi Biotec
CD45 REA747 recombinant human IgG1 1/50 130-110-637 Miltenyi Biotec
PD1 REA1165 recombinant human IgG1 1/50 130-120-391 Miltenyi Biotec
LAG3 REA351 recombinant human IgG1 1/50 130-118-549 Miltenyi Biotec
41BB REA765 recombinant human IgG1 1/50 130-110-763 Miltenyi Biotec
TIM3 REA635 recombinant human IgG1 1/50 130-119-785 Miltenyi Biotec
LNGFR REA844 recombinant human IgG1 1/50 130-112-601 Miltenyi Biotec
LNGFR REA844 recombinant human IgG1 1/50 130-112-602 Miltenyi Biotec
CD4 VIT4 mouse IgG2aκ 1/11 130-120-391 Miltenyi Biotec
CD8 REA734 recombinant human IgG1 1/50 130-110-681 Miltenyi Biotec
CD62L 145/15 mouse IgG1κ 1/50 130-113-621 Miltenyi Biotec
CD45RO REA611 recombinant human IgG1 1/50 130-113-560 Miltenyi Biotec
CD162 REA319 recombinant human IgG1 1/50 130-104-708 Miltenyi Biotec
MACS® Marker Screen various various 1/50 130-110-055 Miltenyi Biotec
8-Color Immunophenotyping Kit, human various recombinant human IgG1 1/50 130-120-640 Miltenyi Biotec

	REA control REA293 recombinant human IgG1 1/50 130-113-450 Miltenyi Biotec<br>REA control REA293 recombinant human IgG1 1/50 130-113-446 Miltenyi Biotec<br>REA control REA293 recombinant human IgG1 1/50 130-113-449 Miltenyi Biotec<br>REA control REA293 recombinant human IgG1 1/50 130-113-454 Miltenyi Biotec<br>REA control REA293 recombinant human IgG1 1/50 130-113-456 Miltenyi Biotec<br>mouse IgG2a control S43.10 mouse IgG2a 1/11 130-113-841 Miltenyi Biotec<br>mouse IgG1 control IS5-21F5 mouse IgG1κ 1/11 130-113-202 Miltenyi Biotec<br>rat IgM control ES26-13D3.4 rat IgMκ 1/11 130-102-672 Miltenyi Biotec<br>Chicken anti-Goat IgG (H+L) Cross-Adsorbed Secondary Antibody, Alexa Fluor 647 polyclonal chicken IgY 10 µg/ml A-21469 ThermoFisher<br>anti-Mouse IgG (Fab specific) antibody polyclonal goat 10 µg/ml M4155-1ML Merck
Validation	We always used target negative and positive cells as controls and isotype controls to ensure antigen dependend staining.<br>Miltenyi Biotec antibodies are validated for use in flow cytometry. We indicated above, as well as in the Supplementary Table T1 and T2 the respective isotypes and species. Specific data about antibody validation can be retrieved from the Miltenyi Biotec webpage (https://www.miltenyibiotec.com/) using the given order numbers.<br>Merck's Anti-Mouse IgG (Fab specific) antibody produced in goat is validated by ELISA, immunoelectro-phoresis (IEP) and Ouchterlony double diffusion (ODD) (https://www.sigmaaldrich.com/catalog/product/sigma/m4155?lang=de&region=DE&cm_sp=Insite-_-prodRecCold_xviews-_-prodRecCold5-3).<br>Thermo Fisher's antibody Chicken anti-Goat IgG (H+L) Cross-Adsorbed Secondary Antibody, Alexa Fluor 647 is validated for immunohistochemnistry and immunofluorescence (https://www.thermofisher.com/antibody/product/Chicken-anti-Goat-IgG-H-L-Cross-Adsorbed-Secondary-Antibody-Polyclonal/A-21469) and rabbit anti-human-CDCP1 is validated for ICC, IF, IP and western blot. (https://www.thermofisher.com/antibody/product/CDCP1-Antibody-Polyclonal/PA5-17245).<br>abcam antibodies: rabbit anti-human-CEACAM6 antibody (ab199277) is validated for IHC (https://www.abcam.com/ceacam6-antibody-ab199277.html). rabbit anti-human-CDCP1 antibody (ab223743) is validated for IHC (https://www.abcam.com/ceacam6-antibody-ab199277.html). rabbit anti-human-TSPAN8 (ab230488) is validated on western blots IHC and flow cytometry (https://www.abcam.com/hexa-antibody-ab230488.html). Southern Biotech antibody mouse Anti-Rabbit IgG-PE is tested for ELISA, FLISA, flow cytometry, IHC and western blot (https://www.southernbiotech.com/?catno=4090-09&type=Monoclonal#&panel1-2&panel2-1).<br>goat anti-rabbit-HRP (414142F) from Medac is validated on IHC (http://www.medac-diagnostika.de/index.php?id_product=3538&controller=product&id_lang=2).<br>Avidin anti-biotin-HRP from (18-4100) from Invitrogen has been validated on ELISA and western blot (https://www.thermofisher.com/document-connect/document-connect.html?url=https%3A%2F%2Fassets.thermofisher.com%2FTFS-Assets%2FLSG%2Fmanuals%2F18-4100.pdf&title=VGVjaG5pY2FsIERhdGEgU2hlZXQ6IEF2aWRpbiBIUlA=).

Eukaryotic cell lines

Policy information about cell lines

Cell line source(s)	Human embryonic kidney 293T (HEK293T, ACC 635) cells were obtained from the DSMZ – German Collection of Microorganisms and Cell Cultures. BxPC3 (CRL-1687) and AsPC1 (CRL-1682) cells were obtained from ATCC and cultured as recommended. PanCa0201 which was derived from a human primary PDAC tumor by the investigators.
Authentication	All cell lines used were authenticated using the Human STR Profiling Cell Authentication Service of ATCC. Exception: PanCa0201 as it is a novel self-generated cell line.
Mycoplasma contamination	not tested
Commonly misidentified lines<br>(See ICLAC register)	None

Animals and other organisms

Policy information about studies involving animals; ARRIVE guidelines recommended for reporting animal research

Laboratory animals	NSG (NOD.Cg-Prkdc<scid>Il2rg<tm1Wjl>SzJ) mice were male, 6-12 weeks old and obtained from Charles River. Mice were kept in IVC cages at room temperature and in a 12-12 light-dark cycle and a humidity between 45 and 65%.
Wild animals	No wild animals were used in this study.
Field-collected samples	No field collected samples were used in this study.
Ethics oversight	All experiments were reviewed and approved by the Landesamt für Natur, Umwelt und Verbraucherschutz NRW, Approval number 84-02.04.2017.A320 and Niedersächsisches Landesamt für Verbraucherschutz und Lebensmittelsicherheit, Approval numbers 33.9-42502-04-13/1085 and 33.9-42502-04-14/1511

Note that full information on the approval of the study protocol must also be provided in the manuscript.

Human research participants

Policy information about studies involving human research participants

Population characteristics	Buffy coats from anonymous healthy donors were purchased from the German Red Cross Dortmund. The researchers were blind to any covariate characteristics.<br>Tumor samples were taken after formal written consent during an excision surgery from patients with pancreatic ductal adenocarcinoma. The researchers were blind to any covariate characteristics.
Recruitment	Surgeons of the Universitätsklinik Göttingen decided based on treatment guidlines, which patient should undergo surgery and samples were forwared to the investigators. The surgeons were not part of the investigators team and were not influenced by their decisions. Thus no self-selection bias could be introduced by investigators.
Ethics oversight	The approved Universitätsmedizin Göttingen Review Board.

Note that full information on the approval of the study protocol must also be provided in the manuscript.

Flow Cytometry

Plots

Confirm that:

- [x] The axis labels state the marker and fluorochrome used (e.g. CD4-FITC).
- [x] The axis scales are clearly visible. Include numbers along axes only for bottom left plot of group (a 'group' is an analysis of identical markers).
- [x] All plots are contour plots with outliers or pseudocolor plots.
- [x] A numerical value for number of cells or percentage (with statistics) is provided.

Methodology

Sample preparation	For flow analysis of PDX models,PDX models were dissociated using the Tumor Dissociation Kit, human in combination with the gentleMACS™ Octo Dissociator with Heaters (both Miltenyi Biotec). Subsequently, mouse cells were depleted using the Mouse Cell Depletion Kit (Miltenyi Biotec). Resulting cell suspensions were analyzed using the MACS® Marker Screen, human (Miltenyi Biotec) a monoclonal antibody panel containing 371 pre-titrated antibodies with 9 isotype controls. All samples were measured on a flow cytometer.<br>Primary PDAC specimen were dissociated and analysed with flow cytomotry as PDX models.<br>Peripheral blood mononuclear cells (PBMCs) were isolated by density gradient centrifugation from buffy coats of healthy anonymous donors (German Red Cross Dortmund). T cells were purified from PBMCs using the Pan T Cell Isolation Kit, human (Miltenyi Biotec) and activated in TexMACS™ Medium (Miltenyi Biotec) containing T Cell TransAct™, human (Miltenyi Biotec) and 100 IU/ml of recombinant Human IL-2 IS, research grade (IL-2) (Miltenyi Biotec). T cells were transduced 24 h after activation using vesicular stomatitis virus glycoprotein G (VSV-G) pseudotyped lentiviral supernatants derived from transfected HEK293T cells. Supernatants were concentrated and stored at -70°C until transduction. 3 d post activation, T Cell TransAct™, human was washed out of the medium and T cells were cultured with 100 IU/ml IL-2 containing TexMACS™ Medium. T cells were used for in vitro assyas directly or frozen until further use for in vivo testing 12-14 d after purification from PBMCs. Frozen T cells that were used for in vivo testing were thawed 24 h before injection in TexMACS™ Medium without further supplements. On the day of use, the amount of living CAR T cells was determined using flow cytometry and staining T cells with 7 AAD and anti human LNGFR (both Miltenyi Biotec).
Instrument	MACSQuant® Analyzer 10, Miltenyi Biotec
Software	Collection and analysis: MACSQunatify v 2.11.1731.18902
Cell population abundance	The purity was verified by flow cytometry.
Gating strategy	PDX:Debris was excluded by SSC A/FSC-A gating and dead cells were excluded using PI or 7-AAD. After exclusion of doublets (FSC-a/FSC-H) mouse cells were excluded by gating on hEpCAM+ human tumor cells. One xenograft was stained before with CellTrace™ Violet to distinguish between both samples. The target candidate expression was analysed on hEpCAM+ cells.<br>Primary tumor: Debris, dead cells and doublets were excluded. Target candidate expression was analysed on leukocytes (CD45+), tumor cells (EpCAM+) or double negative cells.<br>T cells: Debris, dead cells and doublets were excluded. CAR T cells were gated in using fluorescently labeled LNGFR antibody followed by cell txype specific gating using labeled antibodies.

- [x] Tick this box to confirm that a figure exemplifying the gating strategy is provided in the Supplementary Information.
